# RURUS SURYAWAN Score: A Novel Scoring System to Predict 30-Day Mortality for Acute Myocardial Infarction Undergoing Primary Percutaneous Coronary Intervention

**DOI:** 10.3390/jcm14051716

**Published:** 2025-03-04

**Authors:** I Gde Rurus Suryawan, Yudi Her Oktaviono, Budi Baktijasa Dharmadjati, Aldhi Pradana Hernugrahanto, Mochamad Yusuf Alsagaff, David Nugraha, Made Edgard Rurus Surya Erlangga, Pandit Bagus Tri Saputra, Ricardo Adrian Nugraha

**Affiliations:** 1 Division of Interventional Cardiology, Department of Cardiology and Vascular Medicine, Faculty of Medicine Universitas Airlangga, Dr. Soetomo General Academic Hospital, Surabaya 60286, Indonesia; yudi.her@fk.unair.ac.id (Y.H.O.); aldhi.pradana@fk.unair.ac.id (A.P.H.); 2 Division of Arrhythmia, Electrophysiology and Pacing, Department of Cardiology and Vascular Medicine, Faculty of Medicine Universitas Airlangga, Dr. Soetomo General Academic Hospital, Surabaya 60286, Indonesia; budi.baktijasa@fk.unair.ac.id; 3 Division of Acute and Intensive Cardiovascular Care, Department of Cardiology and Vascular Medicine, Faculty of Medicine Universitas Airlangga, Dr. Soetomo General Academic Hospital, Surabaya 60286, Indonesia; yusuf_505@fk.unair.ac.id; 4 Department of Cardiology and Vascular Medicine, Faculty of Medicine Universitas Airlangga, Dr. Soetomo General Academic Hospital, Surabaya 60286, Indonesia; david.nugraha-2018@fk.unair.ac.id (D.N.); made.edgard.rurus-2023@fk.unair.ac.id (M.E.R.S.E.); pandit.bagus.tri-2023@fk.unair.ac.id (P.B.T.S.); ricardo.adrian.nugraha-2019@fk.unair.ac.id (R.A.N.)

**Keywords:** acute myocardial infarction, 30-day mortality, percutaneous coronary intervention, predictor

## Abstract

**Background/Objectives**: It is essential to identify acute myocardial infarction patients with greater risk of deterioration following primary percutaneous coronary intervention. Due to an inconsistent result about predictors of 30-day outcomes regarding scoring systems for the first episode of acute myocardial infarction, the objective of this study is to develop novel scoring systems to predict 30-day mortality among patients with a first episode of acute myocardial infarction who underwent primary percutaneous coronary intervention. **Methods**: This retrospective study was conducted with total sampling for all patients with first-time acute myocardial infarction who underwent primary percutaneous coronary intervention between 2021 and 2024 at Dr. Soetomo Hospital, Indonesia. We performed a total sampling and collected 1714 patients, of which 1535 patients were included. Our primary outcomes included 30-day mortality. **Results**: The analysis included 1535 patients: 926 in the derivation set and 609 in the validation set. In our study, the 30-day mortality rate was 20.7%. Multivariate logistic regression analysis was used to build prediction models in the derivation group and then validated in the validation cohort. The area under the ROC curve of the RURUS SURYAWAN score to predict 30-day mortality was 0.944 (0.906–0.972) in the derivation set and 0.959 (0.921–0.983) in the validation set, with 94.6% sensitivity and 97.3% specificity (*p* < 0.001). **Conclusions**: After adjusting for potential confounders, we developed RURUS SURYAWAN, a novel scoring system to identify predictors of 30-day mortality among acute myocardial infarction before primary percutaneous coronary intervention.

## 1. Introduction

The mortality rate of acute myocardial infarction has significantly decreased in the last decade due to the invention of primary percutaneous coronary intervention by Geoffrey Hartzler in the early 1980s [1]. The prognosis of acute myocardial infarction after the invention of primary percutaneous coronary intervention is better [2]. Every time earlier, reperfusion treatment results in an improvement in mortality rate, and primary percutaneous coronary intervention is still a treatment of choice for acute myocardial infarction [3]. Almost all reports showed a consequent reduction in mortality in acute myocardial infarction patients compared with thrombolysis, which was insufficient to assess the extent of the improvement [4]. Unfortunately, according to several reports, residual 30-day mortality among patients with acute myocardial infarction who underwent primary percutaneous coronary intervention is still high, ranging from 5–10%, especially in developing countries [5]. Data conducted by Pramudyo et al. (2022) revealed that Indonesia ranked fourth in the world for the highest mortality rate of acute myocardial infarction patients compared to 186 other countries [6]. The 30-day mortality rate of acute myocardial infarction in Indonesia was approximately 10.6% for patients with acute myocardial infarction [6]. Still, this number is higher when compared to the mortality rate in other Asian countries (5%), as well as European (4–8%) and American (6–9%) countries [7,8,9].

The high mortality rate of acute myocardial infarction in Indonesia has attracted much attention from cardiologists and is influenced by many factors, such as demographic factors, a history of cardiovascular risk factors, clinical presentation, and the results of investigations and management in the hospital [10]. The high mortality rate in patients with acute myocardial infarction has also raised particular concerns for cardiologists to develop a method to accurately identify patients who are at high risk so that awareness can be increased to reduce the risk of death [11,12]. Over the last 2 decades, there are several prognostic tools developed in the field of acute myocardial infarction: Global Registry of Acute Coronary Events (GRACE), Thrombolysis in Myocardial Infarction (TIMI), Platelet Glycoprotein IIb/IIIa in Unstable Angina: Receptor Suppression Using Integrilin (PURSUIT), Oxford Acute Severity of Illness Score (OASIS), Logistic Organ Dysfunction System (LODS), and Simplified Acute Physiology Score (SAPS II) [13,14,15,16,17,18].

The GRACE score was established to assess 30-day mortality and identify high-risk groups among patients with acute myocardial infarction globally [13]. For global use, the GRACE score demonstrated a superior predictive power for 1-year mortality due to acute myocardial infarction compared with other scoring systems, such as the TIMI and PURSUIT risk scores [14,15]. The TIMI and GRACE risk scores were calculated using data from a Western Caucasian cohort with limited participation from an Asian cohort. Asian patients have been understudied [13,16].

OASIS was proposed in 2013 by machine-learning algorithms. This scoring system has achieved better predictive models for ICU mortality with an area under the receiver operating characteristic curve of 0.88 with only 10 simplified parameters [17]. Unfortunately, this machine-learning technique requires the collection of numerous physiologic measurements, known as particle swarm optimization, making their applications in clinical practice difficult [17]. In China, Wang et al. (2021) introduced a 30-day mortality predictor for ST-Elevation Myocardial Infarction (STEMI) patients known as the LODS scoring system [18]. LODS is an organ dysfunction-based scoring system that should be calculated within the first 24 h of admission. LODS permits the calculation of predicted mortality based on the organ dysfunction score on the day of ICU admission [18]. With a more concise composition of the local population and greater clinical benefit, LODS may be a better predictor of 30-day mortality for intensive care patients with STEMI in China [19].

As many countries developed their own scoring system based on their own data, Indonesia still does not have a local registry nor a scoring system for predictors of 30-day mortality among patients with acute myocardial infarction or acute coronary syndrome [19,20,21]. Since Indonesia had higher mortality for acute myocardial infarction compared to other Asian or Western countries, existing scoring systems were found to be insufficient in predicting 30-day mortality among our local patients. Patient clinical characteristics, genetics, local cultures, and centre performances may affect the results and may even cause the scoring system to overestimate the mortality rate. An accurate scoring system could guide the decisions of treatment in clinical practice and improve the prognosis. For example, the clinical strategies for acute myocardial infarction patients have changed dramatically over a relatively long time period (from 2008 to 2019), from early complete revascularization of all vessels to culprit vessel-only revascularization, indication for CABG and potent antiplatelet, etc. Thus, the aim of this study is to develop a novel scoring system to predict 30-day mortality of patients with firstly diagnosed acute myocardial infarction after primary percutaneous coronary intervention at Dr. Soetomo General Academic Hospital, Surabaya, Indonesia based on our own data and variables, which are obtained before Primary Percutaneous Coronary Intervention (PCI).

## 2. Materials and Methods

### 2.1. Study Population and Design

This retrospective study was conducted at Dr. Soetomo General Academic Hospital, Surabaya, as a tertiary referral hospital in Indonesia from 2021 to 2024. The data collected included the baseline demographics, clinical characteristics, comorbidities, laboratory, echocardiography, and angiography parameters, and we compared it with 30-day mortality rate of first episode of acute myocardial infarction for patients who underwent primary percutaneous coronary intervention during hospital admission. This study has been approved by the Health Research Ethics Committee of the Dr. Soetomo General Academic Hospital, Surabaya. The study was conducted according to the guidelines of the Declaration of Helsinki. The patients in the database are anonymous.

### 2.2. Inclusion and Exclusion Criteria

The population of this study consisted of hospitalized patients with first episode of acute myocardial infarction who underwent primary percutaneous coronary intervention at Dr. Soetomo General Academic Hospital, Surabaya. The inclusion criteria were: (a) adults aged above 18 years; (b) those admitted to the hospital for the first time with emergency causes or complications from first episode of acute myocardial infarction; (c) those who underwent primary percutaneous coronary intervention with total or subtotal occlusion coronary artery from cardiac catheterization; (d) those willing to participate in the study by signing the informed consent form; (e) those who underwent anamnesis and had 50 mL blood taken for laboratory examination; and (f) those able to be followed up at least 1 month after hospitalization. The exclusion criteria were: (a) those admitted for elective procedures; (b) presence of other unrelated diseases such as primary valvular heart disease, congenital heart disease, liver cirrhosis, malignancy, tuberculosis, or HIV/AIDS; (c) history of mechanical circulatory support such as intra-aortic balloon pump or extracorporeal membrane oxygenation; (d) special or vulnerable patients such as prisoners or homeless individuals; and (e) history of cardiac arrest before primary percutaneous coronary intervention.

### 2.3. Statistical Analysis

Statistical analyses were performed using IBM SPSS Statistics for Windows Operating System, version 26.0 software (IBM Corp., Armonk, NY, USA), STATA software, version 15.0 (Stata Corporation, College Station, TX, USA), and R statistics 4.0.3 with the “glmnet” package (R Foundation for Statistical Computing, Vienna, Austria). Baseline characteristics such as gender, age, and categories were described as a proportion if the data were categorical and described as mean or median. Clinical characteristics and comorbidities, such as laboratory, echocardiography, and angiography parameters, were tested using the Multivariate Cox Regression test. A *p*-value below 0.05 was declared statistically significant. The scoring system was assessed to develop the score for each predictor variable contributing to 30-day mortality. To avoid the occurrence of intercorrelation among two or more independent variables, multicollinearity was assessed by examining tolerance and the Variance Inflation Factor (VIF). Primary outcome was 30-day mortality. The score was calculated using the beta coefficient and standard error. The predictive values of our novel risk score were assessed by receiver operating characteristic curve (ROC) analysis (using MedCalc Version 12.2.1; MedCalc Software, Mariakerke, Belgium), applying net reclassification and integrated discrimination improvement. Prognostic utility of the risk models for 30-day mortality has been assessed by deriving their C-statistics, using ROC curves. The final scores were classified based on the risk of acute myocardial infarction patients with high, moderate, and low mortality rates.

In the derivation set, we performed multivariable logistic regression model to determine predictors for 30-day mortality. We use the Least Absolute Shrinkage and Selection Operator (LASSO) logistic regression algorithm in order to obtain a subset of predictor variables from the 20 candidate variables. The LASSO algorithm can select from the set of candidate variables that achieve greater importance once regularized. The LASSO algorithm finds the variables that contribute the least in the logistic regression model and forces them to have coefficients equal to zero. A prior LASSO (pLASSO) was also conducted to incorporate prior information into the generalized linear models and to improve the prediction accuracy for each variable, which were also identified as statistically significant variables using multivariate likelihood ratio (LR). We have removed multicollinear predictors before LASSO to lessen the variance inflation of the standard errors of our variables. For binary variables entered into the multivariate LR model mentioned previously, potential non-linearity in the prediction of 30-day mortality was explored using two-stage prior LASSO (TSPLASSO). To check the stability in the selection of variables and in the calculation of the coefficients of the logistic regression, an analysis with random partitions of the derivation and validation sets was conducted. One hundred sets of derivation have been created randomly with the same number of records as those used in the temporal validation (n  =  926). The LASSO variable selection methodology has been applied to each of these sets, and the corresponding logistic regression coefficients have been calculated. These results were compared with those obtained in the temporal validation.

## 3. Results

This retrospective study evaluates 30-day mortality from 1535 patients assigned to Dr. Soetomo General Academic Hospital with the first episode of acute myocardial infarction and having undergone primary percutaneous coronary intervention during hospital admission. The Hosmer–Lemeshow statistic was 3.56_df8_, (*p* = 0.795). The C statistic for the model in the test cohort was 0.85.

### 3.1. Baseline Characteristics

A total of 1714 patients assigned to Dr. Soetomo General Academic Hospital with the first episode of acute myocardial infarction and having undergone primary percutaneous coronary intervention during hospital admission were screened. All 1714 eligible patients were randomly divided into a derivation (n = 926, 60.33%) and a validation (n = 609, 39.67%) group, according to the grouping method from the previous study (Table 1).

### 3.2. Model Development

For the derivation group, Table 2 shows the variables selected using regression analysis, which were associated with 30-day mortality from univariate analysis. Statistically significant variables screened from the univariate analysis were included in the non-conditional binary multivariate logistic regression. Univariate and multivariate logistic regression identified several variables, as shown in Table 2, as the most significant mortality risk predictors. Heart rate at discharge was related to 1-year mortality after adjustment for these variables (hazard ratio 1.13; 95% confidence interval [CI], 1.03–1.24 per 10 beats per minute, *p* = 0.02). We performed LASSO regression with prior LASSO (pLASSO) to incorporate prior information into the generalized linear models and to improve the prediction accuracy for each variable, which were also identified as statistically significant variables using multivariate likelihood ratio (LR). For binary variables entered into the multivariate LR model mentioned previously, potential non-linearity in the prediction of 30-day mortality was explored using two-stage prior LASSO (TSPLASSO).

### 3.3. RURUS SURYAWAN as Novel Risk Score for 30-Day Mortality Among Acute Myocardial Infarction Patients Undergoing Primary Percutaneous Coronary Intervention

After obtaining results from univariate and multivariate logistic regression analysis, we developed a novel score “RURUS SURYAWAN” to predict 30-day mortality among first-diagnosed acute myocardial infarction patients undergoing primary percutaneous coronary intervention (Table 3).

**Figure 1:**
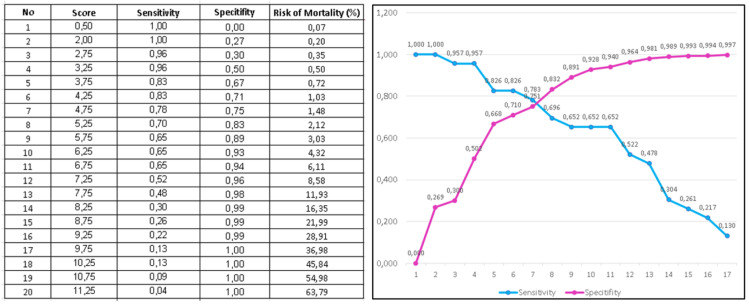
Observed and predicted 30-day mortality based on RURUS SURYAWAN score (maximum 13 points).

The estimated multivariate logistic regression model with the 30-day mortality risk score demonstrated excellent calibration-in-the-large, with an intercept of 0.016. The difference in the log-odds ratio between predictions and observed outcomes in the validation group was statistically significant (*p* = 0.009). When the logistic regression model was recalibrated to the derivation group and applied to subjects in the validation group, it demonstrated good calibration-in-large, and the calibration slope was significantly different (*p* < 0.001). The concordance between the observed probability of 30-day mortality and the predicted probability of 30-day mortality across the 13 vigintiles of variables in the validation sample is described in Figure 1. The Hosmer–Lemeshow X^2^ statistic of the full model was 3.56 (*p* = 0.795).

When assigning a point score, each variable in Table 3 had one point assigned. Therefore, a total of 13 points could be assigned. The score ranged from 0 to 13, and mortality rates increased with a higher score (*p* trend < 0.0001). After grouping patients into low risk (0–3 points), intermediate risk (4–6 points), high risk (7–9 points), and very high risk (10–13 points) in classification groups (Table 4), most deaths occurred in the high-risk and very-high-risk groups. Mortality rates were <1%, 2–5%, 8–30%, and >50% in low-risk, intermediate-risk, high-risk, and very-high-risk groups, respectively (*p* trend < 0.001). This trend persisted when using multiple imputations or a conservative analytical approach, as seen in Figure 1.

## 4. Discussion

We developed and validated the RURUS SURYAWAN score, an improvisation from the previous risk model to predict 30-day mortality for first-diagnosed acute myocardial infarction patients after primary percutaneous coronary intervention. The RURUS SURYAWAN scoring system contains 13 variables, with a score range from 0–13. The final parsimonious scoring systems do not require any collection of sophisticated parameters, making their applications in clinical practice easier and quicker than other scoring systems. This model includes resting heart rate (>110 bpm), underweight (BMI < 18 kg/m^2^), respiratory rate (>28/min), first 24 h urine output (<0.5 mL/kg BW/hour), peripheral O_2_ saturation (<90%), systolic blood pressure during admission (<90 mmHg), urea nitrogen (>50 mg/dL), reduced ejection fraction (<40%), timing of primary percutaneous coronary intervention (door-to-balloon time >2 h), age (>70 years old), sex (women), anemia (Hb < 10 g/dL), and elevated NT-proBNP (>1500 pg/mL) as factors for risk adjustment. The model performed well in an independent validation cohort. A simplified integer score based on this model also performed well and can potentially serve as a foundation for prospective risk stratification at the point of care.

The RURUS SURYAWAN score is stratified into low-risk, intermediate-risk, and high-risk categories based on 13 variables, as mentioned in Table 3. The scores vary from “0-to-13-point scores”. As seen in Table 4, a score of 0–3 indicates a patient is at “low risk” and advises consideration of routine or usual care after primary PCI, and further investigations can be planned during outpatient department visits. Our study saw 30-day mortality for less than 1% of low-risk patients. Patients with a score of 4–6 are classified as “intermediate risk”, and they are usually considered safe but sometimes require monitoring as they have a 2–5% risk of 30-day mortality. Patients with a score of 7–9 are classified as “high risk”, and they require strict clinical observation, including longer duration of intensive care. About 8–30% of high-risk patients had 30-day mortality in our study. Patients with a score of 10–13 are deemed “very high risk” with a more than 50% risk of 30-day mortality and require early aggressive care with very close hemodynamic monitoring during a 30-day stay.

The calculated area under the ROC curve for the RURUS SURYAWAN score to predict 30-day mortality was 0.944 (0.906–0.972) in the derivation set and 0.959 (0.921–0.983) in the validation set, with 94.6% sensitivity and 97.3% specificity (*p* < 0.001). It was non-inferior compared to the existing score. AUROC for the HAS-BLED score was 0.717 (0.680–0.752), with 85.1% sensitivity and 51.5% specificity (*p* < 0.001), whilst AUROC for the TIMI score was 0.844 (0.813–0.871), with a 91.0% sensitivity and 61.6% specificity (*p* < 0.001). AUROC for LODS was 0.867 (0.834–0.895), AUROC for OASIS was 0.827 (0.792–0.859), and AUROC for SAPS II was 0.894 (0.864–0.919), as seen in Table 5 [18,22,23].

In the literature, mostly comparable results were found when comparing the RURUS SURYAWAN score with the HEART score, TIMI score, ACUITY score, CRUSADE score, and GRACE score. In previous study, the AUC of the HEART score was 0.83 (95% CI: 0.81–0.85), the AUC of the TIMI score was 0.844 (95% CI: 0.813–0.871), the AUC of the ACUITY score was 0.72 (95% CI: 0.69–0.75), the AUC of the CRUSADE score was 0.64 (95% CI: 0.61–0.68), and the AUC of the GRACE score was 0.78 (95% CI: 0.75–0.81), which are slightly lower than our AUC of RURUS SURYAWAN score, which was 0.944 (0.906–0.972) in the derivation set and 0.959 (0.921–0.983) in the validation set [22,23]. The TIMI risk score estimates mortality based on: age ≥65, ≥3 coronary artery disease (CAD) risk factors, known CAD (stenosis ≥ 50%), aspirin use in the past 7 days, severe angina (≥2 episodes in 24 h), ST-segment deviation ≥0.5 mm, and positive cardiac markers. The GRACE risk score was calculated by the eight different baseline variables incorporated in the risk calculator: age, heart rate (beats per minute), systolic blood pressure, serum creatinine level, cardiac arrest at admission, ST-segment deviation on ECG, elevated cTn, and congestive heart failure (Killip class).

Scoring systems and prediction models have become essential tools in clinical practice for the stratification of acute myocardial infarction patients. A valid, reliable, and accurate scoring system could help to identify priority and individual criterion scores. Personalized medicine seeks to derive tailored therapies for each acute myocardial infarction patient based on their risk. Any measurable score can be used as a basis to stratify patient risks and guide the treatment. Scoring systems and prediction models are very useful in the physician’s arsenal to facilitate appropriate decision-making processes and to weigh the benefits and risks of any high-risk percutaneous coronary intervention [24], especially to achieve early complete revascularization.

In our retrospective study, several predictors were associated with higher 30-day mortality. Relevant risk factors included vital signs. Vital signs are easy and inexpensive to measure, and when they are measured under controlled conditions, the results are highly reproducible. There are no technical or financial barriers to its incorporation during 30 days of follow-up. Deterioration in vital signs due to acute myocardial infarction is known to indicate a worsened prognosis. Thus, close and intense monitoring, early recognition of deterioration, and appropriate treatment to intervene in vital signs during hospital admission may be beneficial to improve the prognosis of AMI patients. Cardiac arrest has been shown to be a strong important predictor of AMI mortality from several reports [25]. Nevertheless, we decided not to include cardiac arrest since we faced difficulty in adjusting risk for these events due to their heterogeneity in clinical severity, and the inclusion of these patients in hospital scorecards for percutaneous coronary intervention can result in unintended consequences to withhold aggressive treatment.

One of the most important predictors of mortality is the resting heart rate. This study is in line with the study from Jabre, et al. (2014), which revealed that elevated resting heart rate at the time of the acute myocardial infarction identifies patients at increased risk of all-cause and cardiovascular mortality [26]. Even though heart rate measured upon admission is dependent on the sympathetic activity due to chest pain, stress, or anxiety, Jabre et al. (2014) found that resting heart rate upon admission was still a strong prognostic marker of all-cause and cardiovascular mortality for resting heart rate > 110 bpm and cardiovascular mortality only for resting heart rate > 80 bpm during admission [26]. Indeed, resting heart rate might theoretically contribute to increased mortality by increasing myocardial oxygen consumption, thus worsening ischemia, increasing infarct size, and stimulating atherosclerotic progression and plaque instability [27].

Regarding blood urea nitrogen, according to Zhu et al. (2022), blood urea nitrogen was robustly associated with increased short-term mortality in patients with acute myocardial infarction developing cardiogenic shock [28]. Horiuchi et al. (2018) also found that blood urea nitrogen is a strong predictor for 30-day mortality among acute myocardial infarction patients [29].

The inclusion of ejection fraction obtained from transthoracic echocardiography may add significant prognostic information. A previous report from Bosch and Théroux (2005) revealed that adding baseline LVEF into the model improved the prediction of mortality (C statistic 0.73 vs. 0.67) [30].

Individual patient data from four trials (CAPRICORN, EPHESUS, OPTIMAAL, and VALIANT) showed that LVEF is a ubiquitous risk marker associated with death, regardless of the type of AMI. Thus, recent clinical practice guidelines recommended an initial measurement of LVEF for patients with STEMI and NSTEMI because LVEF has both prognostic and therapeutic significance for acute myocardial infarction patients [31]. Unfortunately, significant variability in LVEF measurement rates across clinicians remains.

Additionally, our study results showed that delayed primary percutaneous coronary intervention poses a greater risk of 30-day mortality among patients with first-diagnosed acute myocardial infarction patients. Data from the Thailand PCI registry in 2006 showed the median door-to-device time for AMI patients was 122 min, and the overall mortality rate was 17.0% [32]. Existing studies have also revealed that the impact of short door-to-device time, particularly those smaller than 60 min, on patient mortality remains debatable [33]. Tsukui, et al. (2020) observed that a longer duration of primary PCI > 2 h was significantly linked with 30-day mortality from all causes despite correcting for cardiogenic shock, arrhythmia, or kidney injury [34]. However, shortening door-to-device time < 1 h was not related to mortality from any cause in the multivariate Cox regression analysis [34]. Consistent with our study, in AMI patients treated with primary PCI, door-to-device time longer than 120 min (2 h) was related to 30-day mortality, but a door-to-device time of less than 60 min (1 h) was not associated with a survival benefit (OR 1.89, 95% CI: 1.56–2.37, *p* = 0.002).

Age is one of the most important predictors of survival after acute myocardial infarction. Older patients have a greater risk of 30-day mortality due to recurrent myocardial infarction, heart failure, cardiogenic shock, atrioventricular block, and atrial fibrillation or flutter. In the angiographic substudy of the GUSTO-III trial, older patients had greater risk for TIMI grade 0 flow and lower rates of TIMI grade 3 flow, more multivessel disease, and lower left ventricular ejection fractions [35]. Older patients had their own risk for all-cause mortality due to their anatomic complexity, physiological vulnerability, age-related risks (including prevalent geriatric syndromes), multimorbidity, frailty, disability, cognitive decline, polypharmacy, heterogeneity in life expectancy, and goals of care [36].

Compared to men, women tend to have significantly higher in-hospital mortality for acute myocardial infarction, according to our study. There are several hypothetical reasons why women have poorer prognosis: women who got acute myocardial infarction are generally older during their hospitalization and have higher comorbidity rates than men. Previous reports have suggested that women have longer delays from symptom onset to hospitalization due to several uncertainties in diagnosis and, as a result, tend to have higher 30-day mortality rates for acute myocardial infarction than men [37]. There is one study that revealed that women experiencing acute myocardial infarction died more frequently than men (9.3% vs. 6.1%, *p*  <  0.01) [38].

Anemia is also recognized as a strong, independent risk factor for mortality after acute myocardial infarction. The prevalence of anemia is 28% among acute myocardial infarction patients who underwent primary percutaneous coronary intervention. Our study suggested a significantly increased risk for 30-day mortality in acute myocardial infarction patients with anemia (Hb < 10 g/dL) once various confounding conditions were adjusted for. A previous study revealed that overall mortality in each group containing patients with anemia was higher than in the corresponding non-anemic groups [39]. From the Myocardial Ischemia National Audit Project (MINAP) registry, anemia is independently associated with 30-day (OR 1.28, 95% CI 1.22–1.35) and 1-year mortality (OR 1.31, 95% CI 1.27–1.35), with a reverse J-shaped relationship between hemoglobin levels and mortality outcomes [40]. In the setting of acute myocardial infarction, anemia might worsen ischemia by decreasing the oxygen delivery to the jeopardized myocardium and increasing myocardial oxygen demand due to greater cardiac output to maintain adequate systemic oxygen delivery [41]. Also, patients with anemia are often underprescribed antiplatelet therapy due to bleeding concerns [42].

Lastly, NT-proBNP also has an incremental prognostic value for 30-day mortality over and beyond the TIMI risk score and the GRACE risk calculator. Data concerning optimal timing to measure NT-proBNP during acute myocardial infarction are limited. One of the aforementioned studies, which confirmed the additional value of NT-proBNP, was based on measurements at 24–96 h [43]. Another study revealed that NT-proBNP values upon admission were significantly higher in patients who died compared to those who survived [44]. Thus, it is consistent with our findings that NT-proBNP had a good and comparable predictive value for 30-day mortality.

### 4.1. Strength

As far as we know, the RURUS SURYAWAN score has the strongest AUC with better sensitivity and specificity to discriminate 30-day mortality in the disease-specific cohorts among Asian patients with a first episode of acute myocardial infarction who underwent primary percutaneous coronary intervention during hospital admission. Our study used a combination of derivation and validation feature selection methods. Cross-validation and hyperparameter tuning improved model performance and reduced over-fitting risk. A LASSO regression with prior LASSO (pLASSO) was used to incorporate prior information into the generalized linear models and to improve the prediction accuracy for each variable, which were also identified as statistically significant variables using multivariate likelihood ratio (LR). To ensure the current study’s reliability, all models were validated using untouched validation data that were not used for model construction. We expect our score to be further externally validated in different databases, allowing for additional comparison with other scores.

### 4.2. Limitation

There are certain limitations to our current study. Firstly, the RURUS SURYAWAN score that we developed was for predicting 30-day mortality. The scores have not been validated for predicting mortality within different time frames. Secondly, it requires the collection of variables during admission, making their applications in clinical practice difficult. We attempted to mitigate this effect by using easily obtained variables in the emergency ward. On the other hand, we recognized that several missing variables could result in a skewed outcome. We try to handle missing data by pairwise deletion. Other potential limitations of this study should be acknowledged. Since this research is based on a single-centre retrospective study, this study inevitably has a particular selection bias. We believed that our limitation of a single-centre study might have been contradicted when tested in other settings, thus limiting its indication to be applied outside of our centre. Fortunately, since Dr. Soetomo General Academic Hospital is the biggest referral hospital for the eastern part of Indonesia, we did believe that our subjects were representable enough to be implemented as a novel scoring system for Indonesian patients. Therefore, further studies with larger samples and multicentre cohort should be done to test the external validity of our novel scoring system and to enhance generalizability once our manuscript has been published. We expect that subsequent investigations conducted in the multicentre study will corroborate our findings.

## 5. Conclusions

We developed a RURUS SURYAWAN score, a scoring system to predict risks of 30-day mortality among patients diagnosed with acute myocardial infarction. The variables are easily obtainable upon admission and do not require complex examinations to be observed before primary percutaneous coronary intervention. This risk model for 30-day mortality could evaluate interventions to improve the outcomes of patients with acute myocardial infarction. Although it has achieved adequate internal validation, it must be externally validated.

## Figures and Tables

**Table 1:** Baseline characteristics of study participants.

Variable	Total (n = 1535)	Derivation Group (n = 926)	Validation Group (n = 609)	*p*-Value for Derivation vs. Validation	Alive (n = 1217)	Died (n = 318)	*p*-Value for Alive vs. Dead
Sex				0.856			0.041
Male (n, %)	1211 (78.9%)	730 (78.8%)	481 (78.9%)		972 (79.9%)	239 (75.2%)	
Female (n, %)	324 (21.1%)	196 (21.2%)	128 (21.1%)		245 (20.1%)	79 (24.8%)	
Age, mean ± SD, years	69.86 ± 9.47	69.85 ± 9.49	69.87 ± 9.23	0.798	68.21 ± 9.45	71.42 ± 10.27	0.021
BMI, mean ± SD, kg/m^2^	23.12 ± 6.93	23.05 ± 8.23	23.29 ± 7.25	0.767	24.27 ± 8.03	21.43 ± 7.56	0.033
Resting heart rate during admission, mean ± SD, bpm	91.44 ± 11.03	91.43 ± 11.97	91.45 ± 12.05	0.863	88.75 ± 10.99	96.82 ± 17.49	0.024
Systolic blood pressure during admission, mean ± SD, mmHg	123.37 ± 14.69	123.71 ± 13.18	123.33 ± 13.95	0.512	127.51 ± 13.32	112.56 ± 21.44	<0.001
Diastolic blood pressure during admission, mean ± SD, mmHg	64.27 ± 9.68	64.64 ± 9.50	64.30 ± 9.12	0.918	64.90 ± 7.90	61.87 ± 12.88	0.053
Respiratory rate during admission, mean ± SD, bpm	22.80 ± 4.10	22.82 ± 3.99	22.76 ± 4.03	0.821	20.62 ± 3.90	28.56 ± 7.84	0.039
Core temperature during admission, mean ± SD, °C	36.23 ± 0.72	36.24 ± 0.71	36.22 ± 0.78	0.719	36.40 ± 0.52	36.14 ± 0.87	0.125
Saturation O_2_ peripheral, mean ± SD, %	97.31 ± 4.87	97.24 ± 4.67	97.35 ± 4.19	0.658	97.63 ± 3.56	92.23 ± 6.34	0.026
Urine-output (first 24 h), median (lower-upper), mL/24 h	1.350 (880–2.200)	1.340 (870–2.100)	1.355 (886–2.230)	0.932	1.480 (890–2.300)	1.160 (550–1.900)	<0.001
LVEF, mean ± SD, %	46.93 ± 13.93	46.92 ± 13.75	46.88 ± 14.27	0.799	49.32 ± 11.77	41.27 ± 21.76	0.042
Haematocrit, mean (SD), %	31.92 ± 5.20	31.77 ± 5.13	32.23 ± 5.36	0.151	31.92 ± 5.19	31.67 ± 5.27	0.571
Red cells, mean (SD), ×10^12^/L	3.59 ± 0.63	3.55 ± 0.62	3.63 ± 0.65	0.088	3.57 ± 0.62	3.55 ± 0.70	0.411
MCH, mean (SD), pg	27.87 ± 1.55	27.85 ± 1.49	27.89 ± 1.69	0.394	29.54 ± 2.58	29.61 ± 2.87	0.647
MCHC, mean (SD), %	29.81 ± 1.34	29.87 ± 1.37	29.77 ± 1.35	0.701	29.88 ± 1.41	29.75 ± 1.36	0.275
MCV, mean (SD), fL	89.91 ± 6.53	90.03 ± 6.47	89.63 ± 6.67	0.396	89.81 ± 6.43	90.47 ± 7.16	0.242
RDW, mean (SD), %	15.96 ± 2.13	16.03 ± 2.25	15.83 ± 1.82	0.139	15.83 ± 2.07	16.75 ± 2.33	<0.001
White cells, mean (SD), ×10^9^/L	10.55 ± 4.29	10.57 ± 4.31	10.54 ± 4.23	0.980	10.20 ± 4.11	12.78 ± 4.78	<0.001
Platelet count, median (lower-upper), ×10^9^/L	222.67 (168.91–304.25)	222.12 (168.91–301.38)	224.03 (168.97–306.78)	0.582	226.09 (173.40–305.33)	198.56 (134.68–262.07)	<0.001
NT-proBNP, median (lower-upper), pg/mL	5840.00 (2251.00–14,968.00)	6217.00 (2341.00–15,555.00)	4994.00 (2088.38–13,629.75)	0.102	5302.00 (2143.00–13,666.50)	9469.00 (9082.50–10,662.75)	<0.001
hsTnI, median (lower-upper), ng/mL	89.25 (46.00–185.19)	85.00 (46.92–185.88)	99.00 (44.00–185.00)	0.509	90.00 (47.00–182.13)	83.12 (37.85–38.63)	0.794
Blood urea nitrogen, median (lower-upper), mg/dL	30.67 (20.83–45.25)	30.67 (20.56–45.08)	30.49 (22.25–45.36)	0.580	29.25 (20.11–43.00)	39.62 (27.06–57.16)	<0.001
Random blood glucose, mean (SD), mEq/L	148.82 ± 51.49	148.09 ± 52.09	150.43 ± 50.12	0.461	148.12 ± 50.46	153.03 ± 57.63	0.271
Sodium, mean (SD), mEq/L	138.87 ± 5.15	138.85 ± 5.17	138.91 ± 5.12	0.536	139.03 ± 4.98	138.18 ± 6.02	0.023
Potassium, mean (SD), mEq/L	4.17 ± 0.71	4.16 ± 0.71	4.18 ± 0.72	0.662	4.11 ± 0.69	4.52 ± 0.83	<0.001
Chloride, mean (SD), mEq/L	102.29 ± 5.24	102.17 ± 5.73	102.42 ± 5.54	0.775	102.57 ± 5.61	103.07 ± 6.10	0.140
Calcium, total, mean (SD), mg/dL	8.51 ± 0.67	8.49 ± 0.68	8.53 ± 0.66	0.369	8.53 ± 0.65	8.23 ± 0.66	<0.001
Magnesium, mean (SD), mg/dL	2.12 ± 0.25	2.11 ± 0.26	2.13 ± 0.24	0.520	2.17 ± 0.24	2.11 ± 0.29	0.012
pH, mean (SD)	7.37 ± 0.07	7.37 ± 0.06	7.36 ± 0.07	0.241	7.41 ± 0.06	7.33 ± 0.07	<0.001
PO_2_, mean (SD), mm Hg	85.54 ± 12.86	85.48 ± 12.95	85.67 ± 12.65	0.846	86.82 ± 12.85	84.07 ± 12.85	0.141
PCO_2_, mean (SD), mm Hg	45.54 ± 12.86	45.48 ± 12.95	45.67 ± 12.65	0.846	45.82 ± 12.85	44.07 ± 12.85	0.141
Bicarbonate, mean (SD), mEq/L	26.92 ± 5.17	26.91 ± 5.23	26.93 ± 5.02	0.706	27.33 ± 4.98	24.01 ± 5.42	<0.001
Lactate, median (Q1–Q3), mmol/L	1.63 (1.22–2.21)	1.61 (1.20–2.07)	1.65 (1.25–2.23)	0.309	1.57 (1.17–2.08)	2.01 (1.38–3.18)	<0.001
Hypertension				0.772			0.053
No (n, %)	690 (45%)	417 (45.03%)	274 (45%)		574 (47.16%)	116 (36.48%)	
Yes (n, %)	845 (55%)	509 (54.96%)	335 (55%)		643 (52.83%)	202 (63.52%)	
Supraventricular arrhythmias				0.818			<0.001
No (n, %)	1404 (91.4%)	847 (91.47%)	557 (91.46%)		1155 (946.91%)	249 (78.3%)	
Yes (n, %)	131 (8.5%)	79 (8.53%)	52 (8.54%)		62 (5.09%)	69 (21.69%)	
Ventricular arrhythmias				0.757			0.514
No (n, %)	1417 (92.31%)	855 (92.33%)	562 (92.28%)		1123 (92.27%)	291 (91.51%)	
Yes (n, %)	118 (7.68%)	71 (7,67%)	47 (7.71%)		94 (7.72%)	27 (8.49%)	
Diabetes mellitus				0.390			0.086
No (n, %)	681 (57.86%)	484 (58.67%)	197 (55.97%)		579 (56.93%)	102 (64.15%)	
Yes (n, %)	496 (42.14%)	341 (41.33%)	155 (44.03%)		438 (43.07%)	57 (35.85%)	
Anemia				0.822			<0.001
No (n, %)	778 (66.10%)	547 (66.30%)	231 (65.62%)		653 (64.21%)	124 (77.99%)	
Yes (n, %)	399 (33.90%)	278 (33.70%)	121 (34.38%)		364 (35.79%)	35 (22.01%)	
Hyperlipidaemia				0.275			0.067
No (n, %)	730 (62.02%)	520 (63.03%)	210 (59.66%)		620 (60.96%)	109 (68.55%)	
Yes (n, %)	447 (37.98%)	305 (36.97%)	142 (40.34%)		397 (39.04%)	50 (31.45%)	
Chronic kidney disease				0.937			<0.001
No (n, %)	747 (63.47%)	523 (63.39%)	224 (63.64%)		625 (61.46%)	122 (76.73%)	
Yes (n, %)	430 (36.53%)	302 (36.61%)	128 (36.36%)		392 (38.54%)	37 (23.27%)	
Chronic obstructive pulmonary disease				0.697			<0.001
No (n, %)	1389 (90.49%)	761 (92.24%)	327 (92.90%)		1118 (91.86%)	271 (85.22%)	
Yes (n, %)	146 (9.51%)	64 (7.76%)	25 (7.10%)		99 (8.13%)	47 (14.78%)	
30-day mortality				0.106			–
No (n, %)	1217 (79.28%)	709 (76.57%)	508 (83.42%)		–	–	
Yes (n, %)	318 (20.72 %)	217 (23.43%)	101 (16.58 %)		–	–	

**Table 2:** Univariate and multivariate logistic regression analysis variables.

Variables	Univariate Analysis			Multivariate Analysis		
	OR	95% CI	*p* Value	OR	95% CI	*p* Value
Sex, women, %	1.33	1.04–1.78	0.037	1.17	1.02–1.33	0.041
Age, years	1.72	1.34–2.24	0.0197	1.55	1.23–2.08	0.021
BMI, mean ± SD, kg/m^2^	1.44	1.18–2.12	0.027	1.37	1.15–1.99	0.033
Resting heart rate, bpm	1.78	1.48 to 2.14	<0.001	1.51	1.20 to 1.90	0.004
Systolic blood pressure during admission (<90 mmHg)	2.97	1.95–4.59	<0.001	2.31	1.67–4.02	<0.001
Respiratory rate during admission, bpm	1.21	1.04–1.45	0.032	1.18	1.03–1.34	0.039
Saturation O_2_ peripheral (<90%)	1.45	1.28–1.77	0.018	1.35	1.21–1.49	0.026
Urine-output, first 24 h admission (<0.5 mL/kg BW/hour)	2.81	1.98–5.13	<0.001	2.56	1.79–4.44	<0.001
Blood urea nitrogen, mg/dL	2.02	1.91–2.13	<0.001	1.93	1.81–2.04	<0.001
Red blood cells, ×10^12^/L	0.82	0.58–1.14	0.233	NA		
Haemoglobin, g/dL	1.94	1.62–2.88	0.002	1.79	1.45–2.55	0.003
MCV, fL	1.03	0.92–1.98	0.120	NA		
MCH, pg	1.07	0.99–1.16	0.098	NA		
White cells, ×10^9^/L	1.11	0.96–1.16	0.057	NA		
Lymphocytes, %	0.92	0.88–1.15	0.078	NA		
Platelet count, ×10^9^/L	1.12	0.98–1.16	0.074	NA		
PaO_2_, mm Hg	0.91	0.86–1.03	0.355	NA		
PaCO_2_, mm Hg	0.94	0.86–0.97	0.098	NA		
Sodium, mEq/L	0.96	0.91 to 0.99	0.196	NA		
Potassium, mEq/L	1.77	1.23–2.17	0.052	NA		
Calcium, mg/dL	0.95	0.78–1.34	0.144	NA		
Magnesium, mg/dL	0.89	0.27–1.02	0.064	NA		
Anion gap, mEq/L	1.23	1.15–1.32	0.048	1.05	0.97–1.27	0.053
Lactate, mmol/L	1.28	1.18–1.44	0.046	1.07	0.99–1.40	0.052
Chronic kidney disease	No	1.30–2.76	0.016	1.54	1.14–2.43	0.024
	Yes					
hsTnI, ng/mL	1.02	0.95–1.07	0.355	NA		
NT-proBNP, pg/mL	2.82	1.90–4.65	<0.001	2.17	1.58–3.74	<0.001
Ejection Fraction, %	1.45	1.22–1.92	0.028	1.21	1.08–1.37	0.042
Time for Percutaneous Coronary Intervention, minutes	1.91	1.58–2.57	<0.001	1.89	1.56–2.37	0.002

**Table 3:** RURUS SURYAWAN score assignment.

Variables	Multivariate Analysis			Point Assigned
	OR	95% CI	*p*-Value	
**R**esting heart rate (>110 bpm)	1.51	1.20–1.90	0.024	1
**U**nderweight (BMI < 18 kg/m^2^)	1.37	1.15–1.99	0.033	1
**R**espiratory rate (>28/min)	1.18	1.03–1.34	0.039	1
**U**rine-output, first 24 h admission (<0.5 mL/kg BW/hour)	2.56	1.79–4.44	<0.001	1
**S**aturation O_2_ peripheral (<90%)	1.35	1.21–1.49	0.026	1
**S**ystolic blood pressure during admission (<90 mmHg)	2.31	1.67–4.02	<0.001	1
**U**rea nitrogen (>50 mg/dL)	1.93	1.81–2.04	<0.001	1
**R**educed ejection fraction. (baseline LVEF < 40%)	1.21	1.08–1.37	0.042	1
time dela**Y**ed of primary percutaneous coronary intervention (door-to-balloon time > 2 h)	1.89	1.56–2.37	0.002	1
**A**ge (>70 years old)	1.55	1.23–2.08	0.021	1
**W**omen	1.17	1.02–1.33	0.041	1
**A**nemia (Hb < 10 g/dL)	1.79	1.45–2.55	0.003	1
**N**T-proBNP (>1500 pg/mL)	2.17	1.58–3.74	<0.001	1
Total score				13

**Table 4:** RURUS SURYAWAN score stratification.

RURUS SURYAWAN Score	Stratification	Risk of 30-Day Mortality
0–3 points	Low risk	<1%
4–6 points	Intermediate risk	2–5%
7–9 points	High risk	8–30%
10–13 points	Very high risk	>50%

**Table 5:** Comparison of ROC curves between each scoring system.

Scoring System	AUC	95%CI	Sensitivity (%)	Specificity (%)
RURUS SURYAWAN	0.944	0.906–0.972	94.6	97.3
HEART	0.830	0.810–0.850	NA	NA
ACUITY	0.720	0.690–0.750	NA	NA
CRUSADE	0.640	0.610–0.680	NA	NA
GRACE	0.780	0.750–0.810	NA	NA
HAS-BLED	0.717	0.680–0.752	85.1	51.5
TIMI	0.844	0.813–0.871	91.0	61.6
LODS	0.867	0.834–0.895	70.19	87.80
OASIS	0.827	0.792–0.859	67.31	85.89
SAPS II	0.894	0.864–0.919	89.42	74.40

## Data Availability

The data belong to the Division of Interventional Cardiology, Department of Cardiology and Vascular Medicine, Faculty of Medicine Universitas Airlangga—Dr. Soetomo General Academic Hospital and require institutional agreements for data release to third parties. Hence, ethical approval is needed for analysis. However, data are available from the Division of Interventional Cardiology, Department of Cardiology and Vascular Medicine, Faculty of Medicine Universitas Airlangga—Dr. Soetomo General Academic Hospital upon reasonable request by emailing them at kardiologiunair@gmail.com.

## Abbreviations

The following abbreviations are used in this manuscript:

ACS	Acute Coronary Syndrome
AMI	Acute Myocardial Infarction
AUC	Area under the curve
AUROC	Area under the receiver operating characteristic
CAPRICORN	CArvedilol Post-infaRct survIval COntRolled evaluatioN
EPHESUS	Eplerenone in patients with heart failure due to systolic dysfunction complicating acute myocardial infarction
GRACE	Global Registry of Acute Coronary Events
GUSTO	Global Utilization of Streptokinase and Tissue Plasminogen Activator for Occluded Coronary Arteries
ICU	Intensive Care Unit
LASSO	Least Absolute Shrinkage and Selection Operator
MINAP	Myocardial Ischemia National Audit Project
NSTEMI	Non-ST-Elevation Myocardial Infarction
NT-proBNP	N-terminal prohormone of brain natriuretic peptide
OASIS	Oxford Acute Severity of Illness Score
OPTIMAAL	Optimal Therapy in Myocardial Infarction with the Angiotensin II Antagonist Losartan
PCI	Percutaneous Coronary Intervention
PPCI	Primary Percutaneous Coronary Intervention
PURSUIT	Platelet Glycoprotein IIb/IIIa in Unstable Angina: Receptor Suppression Using Integrilin
ROC	Receiver operating characteristic
STEMI	ST-Elevation Myocardial Infarction
TIMI	Thrombolysis in Myocardial Infarction
VALIANT	VALsartan In Acute Myocardial Infarction
VIF	Variance Inflation Factor
